# Impact of socio-economic status on hospital length of stay following injury: a multicenter cohort study

**DOI:** 10.1186/s12913-015-0949-2

**Published:** 2015-07-25

**Authors:** Lynne Moore, Brahim Cisse, Brice Lionel Batomen Kuimi, Henry T. Stelfox, Alexis F. Turgeon, François Lauzier, Julien Clément, Gilles Bourgeois

**Affiliations:** Department of social and preventive medicine, Laval University, Quebec, QC Canada; Population Health and Optimal Health Practices Research Unit, Trauma - Emergency - Critical Care Medicine, Centre de Recherche du Centre Hospitalier Universitaire de Québec (CHU de Québec - Hôpital de l’Enfant-Jésus), Laval University, Québec, QC Canada; Department of Critical Care Medicine, Medicine and Community Health Sciences, Institute for Public Health, University of Calgary, Calgary, AB Canada; Department of Anesthesiology and Critical Care Medicine, Division of Critical Care Medicine, Laval University, Québec, QC Canada; Department of Medicine, Laval University, Québec, QC Canada; Department of Surgery, Laval University, Québec, QC Canada; Institut National d’Excellence en Santé et en Services Sociaux, Montréal, Qc Canada

**Keywords:** Injury, Hospital length of stay, Socioeconomic status, Material deprivation, Social deprivation

## Abstract

**Background:**

Injury is second only to cardiovascular disease in terms of acute care costs in North America. One key to improving injury care efficiency is to generate knowledge on the determinants of resource use. Socio-economic status (SES) is a documented risk factor for injury severity and mortality but its impact on length of stay (LOS) for injury admissions is unknown. This study aimed to examine the relationship between SES and LOS following injury.

This multicenter retrospective cohort study was based on adults discharged alive from any trauma center (2007–2012; 57 hospitals; 65,486 patients) in a Canadian integrated provincial trauma system. SES was determined using ecological indices of material and social deprivation. Mean differences in LOS adjusted for age, gender, comorbidities, and injury severity were generated using multivariate linear regression.

**Results:**

Mean LOS was 13.5 days. Patients in the highest quintile of material/social deprivation had a mean LOS 0.5 days (95 % CI 0.1-0.9)/1.4 days (1.1-1.8) longer than those in the lowest quintile. Patients in the highest quintiles of both social and material deprivation had a mean LOS 2.6 days (1.8-3.5) longer than those in the lowest quintiles.

**Conclusions:**

Results suggest that patients admitted for traumatic injury who suffer from high social and/or material deprivation have longer acute care LOS in a universal-access health care system. The reasons behind observed differences need to be further explored but may indicate that discharge planning should take patient SES into consideration.

## Background

Each year, approximately 212,000 Canadians are hospitalized following injury, 68,000 are permanently impaired and 14,000 die with direct costs estimated at 20 $ billion [1]. Trauma care is one of the most resource-intensive medical specialties and second only to cardiovascular diseases in terms of health care costs [1]. Unnecessary hospital days represent an estimated 20 % of total length of stay (LOS) in acute care hospitals [2] implying an important waste of resources as well as increased patient exposure to adverse events and functional decline [3–5]. Reducing LOS has been identified as one of the core strategies for alleviating health care financial pressure and improving patient outcome [6]. Other than poor quality of care, many factors are now being considered as drivers of unnecessarily prolonged hospital stays, including socio-economic status (SES) [6].

The impact of SES on LOS related to general admissions [7] or specific diagnoses such as stroke [8], pneumonia [9, 10], and psychiatric conditions [11] has been widely discussed in the literature [12–15]. In addition, low SES has been consistently documented as a risk factor for injury-related mortality, injury hospitalizations, and injury severity [16]. However, compared to chronic disease populations, injury admissions represent younger patients with fewer comorbidities, different discharge patterns and specific socio-economic issues [17]. In order to improve the quality and efficiency of acute injury care, we need to improve our understanding of the influence of SES on LOS for injury admissions.

The objective of this study was to examine the relationship between SES and hospital LOS following an acute care admission for injury.

## Methods

### Study Population

This retrospective, multicenter cohort study was based on the integrated and mature trauma system in Québec, Canada, instated in 1993. The trauma system comprises a network of specialized acute care centers from level I (highly specialized urban centers with neurosurgical coverage 24/7) to level IV (small rural community hospitals). The system currently comprises 59 hospitals designated according to American College of Surgeons criteria including 5 level I (of which 2 are pediatric), 5 level II, 21 level III, and 28 level IV centers. All level I centers are trauma teaching hospitals.

The study population was identified using the provincial trauma registry and included all adults (≥16 years old) hospitalized between April 1, 2007 and February 28, 2012 with a principal diagnosis of injury (International Classification of Diseases, version 9 codes 800–859 excluding late effects and complications, foreign bodies, poisoning, drowning and burns) admitted to any of the trauma system’s 57 adult trauma centers according to the following trauma registry inclusion criteria: length of stay > 3 days, intensive care unit admission, or transfer from another hospital. In-hospital deaths as well as patients aged 65 years or older with isolated hip fractures and no other major injuries were excluded [18]. The latter are excluded because isolated hip fractures are widely considered to be the consequence of chronic disease [18] and these patients are often treated in hospitals outside the trauma system.

### Study data sources

The trauma registry is maintained through an application housed in each trauma center and connected to a unique central database located at the Ministry of Health. Data collection is mandatory for all patients meeting the inclusion criteria described above. To ensure the reliability and validity of data in the registry, the Ministry of Health conducts regular audits to identify and correct aberrant data values in all data fields and to verify date and time chronology.

### Primary outcome

The primary outcome was acute care LOS, calculated as the number of days between admission and discharge.

### Socio-economic status

SES was quantified using ecological indices of social and material deprivation derived and validated previously using Canadian census data [19, 20] and based on the work by Townsend [21]. These indices are based on the smallest geostatistical unit used in the Canadian census (400 to 700 persons on average) and defined by patients’ postal code [1, 20]. Material deprivation encompasses education (proportion without a high school diploma), employment (the employment/population ratio), and income (average household income). Social deprivation is based on the proportion of people separated, divorced, or widowed; living alone; and single-parent families. The two composite indices were derived using principal components analysis, standardized for age and sex, and divided into quintiles [16]. Patients in the highest quintile are those suffering from the greatest material/social deprivation. This ecological approach is widely used as a proxy for individual SES data [1, 16, 19, 22] and the indices used in this study have been used to evaluate the influence of SES on health outcomes in several Canadian cohort studies [23–26]. We explored the effect of material and social deprivation individually and the interaction between the two. For the latter, patients in the highest quintile of material and social deprivation were compared to those in the lowest quintile for both indices [20].

### Statistical analysis

We used a mixed linear model to estimate mean differences in hospital LOS across SES categories adjusted for physiological reserve, anatomical injury severity, physiological reaction to injury, and transfer status. Physiological reserve was described using age, gender and the number of comorbidities, according to Charlson’s classification [27]. Anatomical injury severity was described by the mechanism of injury, body region of the most severe injury and the maximum Abbreviated Injury Scale score (AIS) [28]. The AIS is a lexicon describing anatomical injuries, each one accompanied by a severity grade from 1 (least severe) to 6 (most severe), established by expert consensus. Physiological response to injury was quantified using the Glasgow Coma Scale (GCS) score [29], a measure of state of consciousness from 3 (no reaction) to 15 (fully alert), systolic blood pressure (SBP) and respiratory rate (RR), all measured on arrival at the emergency department. Independent variables were modelled as dummy variables on categories, as specified in Table 1. A random intercept on hospital was used to control for clustering by trauma center. We did not adjust for health care payer, residential remoteness, or discharge destination as these factors are considered to be mediators in the SES-LOS association and/or strong proxies for SES [30]. Based on evidence in the literature, we hypothesized, à priori, that the influence of social/material deprivation on LOS would differ for young and geriatric patients [31]. Analyses were therefore performed for the whole study sample and according to age group (<65, ≥ 65 years old). Note that we chose a simple linear model over more complex models (e.g., log-linear or gamma models) because arithmetic means have been shown to be an unbiased and efficient estimator of the mean for skewed data given large sample sizes [32, 33], mean differences are more intuitive than geometric mean ratios and sensitivity analysis showed that using more complex models did not change study conclusions. LOS >120 days were truncated at 120 days [34].

**Table 1:** Description of the study population according to the highest levels of material and social deprivation

Characteristics of the study population n (%)		Whole study population	Material deprivation Quintile 5	Social deprivation Quintile 5
Total		65,486	17,172 (26.3)	13,247 (20.2)
Age^e^	16-54	26,240 (40.1)	7527 (28.7)	4709 (18.0)
	55-64	10,054 (15.3)	2698 (26.8)	1839 (18.3)
	65-74	8685 (13.3)	2255 (26.0)	1749 (20.1)
	75-84	11,931 (18.2)	2765 (23.2)	2788 (23.4)
	≥85	8576 (13.1)	1927 (22.5)	2162 (25.2)
Gender^e^	Male	34,031 (52.0)	9329 (27.4)	6105 (17.9)
	Female	31,455 (48.0)	7843 (24.9)	7142 (22.7)
Number of comorbidities	0	43,269 (66.1)	11,554 (26.7)	8007 (18.5)
	1	12,442 (19.0)	3168 (25.5)	2817 (22.6)
	2	5844 (8.9)	1473 (25.2)	1433 (24.5)
	≥3	3931 (6.0)	977 (24.9)	990 (25.2)
Mechanism of injury	Motor vehicle collision	14,707 (22.4)	4143 (28.2)	2244 (15.3)
	Fall	41,170 (62.9)	10,072 (24.5)	9202 (22.4)
	Penetrating	2226 (3.4)	774 (34.8)	495 (22.2)
	Other	7383 (11.3)	2183 (30.0)	1306 (17.7)
Maximum abbreviated injury scale score	1-2	26,894 (41.1)	7428 (27.6)	5505 (20.5)
	3	28,271 (43.2)	7136 (25.2)	5681 (20.1)
	4	6787 (10.3)	1767 (26.0)	1251 (18.4)
	5-6	3534 (5.4)	841 (23.8)	810 (22.9)
Body Region, most severe injury	Head	12,749 (19.5)	3333 (26.1)	2783 (21.8)
	Thorax	9096 (13.9)	2486 (27.3)	1663 (18.3)
	Abdomen	1854 (2.8)	528 (28.5)	378 (20.4)
	Spine	6784 (10.4)	1687 (24.9)	1263 (18.6)
	Upper extremities	11,274 (17.2)	3041 (27.0)	2366 (21.0)
	Lower extremities	23729 (36.2)	6097 (25.7)	4794 (20.2)
Glasgow coma scale^f^	3-8	3358 (5.1)	949 (28.3)	602 (17.9)
	9-12	2010 (3.1)	512 (25.5)	459 (22.8)
	13-15	60,118 (91.8)	15,711 (26.1)	12,186 (20.3)
Systolic blood pressure^f^	Normal (≥90)	64,302 (98.2)	16842 (26.2)	13,012 (20.2)
	Shock (0–89)	1184 (1.8)	330 (27.9)	235 (19.9)
Respiration rate^f^	Normal (10–29)	63,869 (97.5)	16,732 (26.2)	12,934 (20.3)
	Abnormal (0–10; ≥30)	1617 (2.5)	440 (27.2)	313 (19.4)
Transfer-in	No	44,526 (68.0)	10,190 (23.0)	9958 (22.4)
	Yes	20,960 (32.0)	6982 (33.3)	3289 (15.7)
Health care payer	Provincial public	43,771 (66.9)	11,794 (26.9)	9210 (21.0)
	Road accidents	11,282 (17.2)	3019 (26.8)	1954 (17.3)
	Work accidents	2903 (4.4)	868 (29.9)	401 (13.8)
	Other	3586 (5.5)	679 (18.9)	744 (20.8)
	None/Unknown	3944 (6.0)	812 (20.6)	938 (23.8)
Residential remoteness	Metropolitan Region^a^	21,282 (32.5)	2969 (14.0)	5975 (28.1)
	Other Regions^b^	11,214 (17.1)	1807 (16.1)	3065 (27.3)
	Agglomerations^c^	11,824 (18.1)	2654 (22.5)	3328 (28.2)
	Small towns & rural areas^d^	21,166 (32.3)	9742 (46.0)	879 (4.2)
Discharge destination	Home	38,276 (58.5)	10,230 (26.7)	6960 (18.2)
	Long stay	4264 (6.5)	806 (18.9)	1146 (26.9)
	Rehab	6145 (9.4)	1313 (21.4)	1500 (24.4)
	Acute care	5531 (8.5)	1842 (33.3)	1080 (19.5)
	Other	11,270 (17.1)	2981 (26.5)	2493 (22.6)

^a^population size: > 1,000,000

^b^population size: 100,000 – 1,000,000

^c^population size: 10,000 –100,000

^d^population size: < 10,000

^e^Deprivation index is standardized for age and sex

^f^On arrival

The GCS, RR, and SBP were missing for 57 %, 33 % and 12 % of data observations, respectively. As previously described, these data were mostly missing in patients with minor extracranial injury [35]. Missing data were simulated using multiple imputation. The Markov Chain Monte Carlo method was used with a non-informative prior and a single chain to generate five imputes for each missing data value [35, 36]. The imputation model included all independent and dependant variables used in the analyses models.

### Sensitivity analyses

We used sensitivity analyses to evaluate the robustness of study results to the exclusion of deaths, GCS/SBP/RR simulated by multiple imputation and additional hospital days due to transfer. Risk-adjusted mean differences in LOS for SES quintiles in the original analysis were thus compared to those generated by models with i) deaths included but attributed the maximum observed LOS (120 days) [37], ii) observations with missing GCS/SBP/RR excluded, and iii) index LOS replaced with total LOS for all consecutive hospital admissions for the same injury. For the latter, we used trauma registry data linked to hospital administrative discharge data, as described elsewhere [30].

All analyses were performed using SAS software (Version 9.4 of the SAS System for Windows, Copyright ©, SAS Institute Inc., Cary, NC, USA) and statistical tests were two-sided with statistical significance set at 5 %. Ethics approval for this study was obtained from the Comité d’éthique de la recherche avec des êtres humains de l’Université Laval and the Comité d’éthique du Centre Hospitalier Universitaire de Québec.

## Results

Between April 1, 2007 and February 28, 2012, 72,009 patients were eligible for the study. A deprivation index couldn’t be assigned to 2686 patients (3.7 %) due to a missing or invalid postal code and to 3837 patients (5.3 %) because no SES information was available for their residential zone (i.e., patients living in a high density long term care/ institutional facilities or in a rural community with very low density population) [19]. The final study population therefore comprised 65,486 patients.

Over 40 % of the study population was aged ≥ 65 years, over one half were male and one fifth of patients were admitted for major trauma (Injury Severity Score > 15). Social deprivation increased with age and was more prevalent in patients with multiple comorbidities whereas material deprivation was lower for elderly patients (Table 1). Material deprivation was higher in males than in females, but we observed the opposite for social deprivation. However, the associations between age/gender and SES indices are difficult to interpret as the latter are standardized for age and gender. Material deprivation was more prevalent in patients with penetrating injuries but decreased with increasing injury severity. Social deprivation increased with increasing injury severity but was less prevalent in patients covered by work accident insurance than those covered by other health care payers. Patients residing in metropolitan regions suffered from higher social deprivation but lower material deprivation than patients residing in rural areas.

**Fig. 1:**
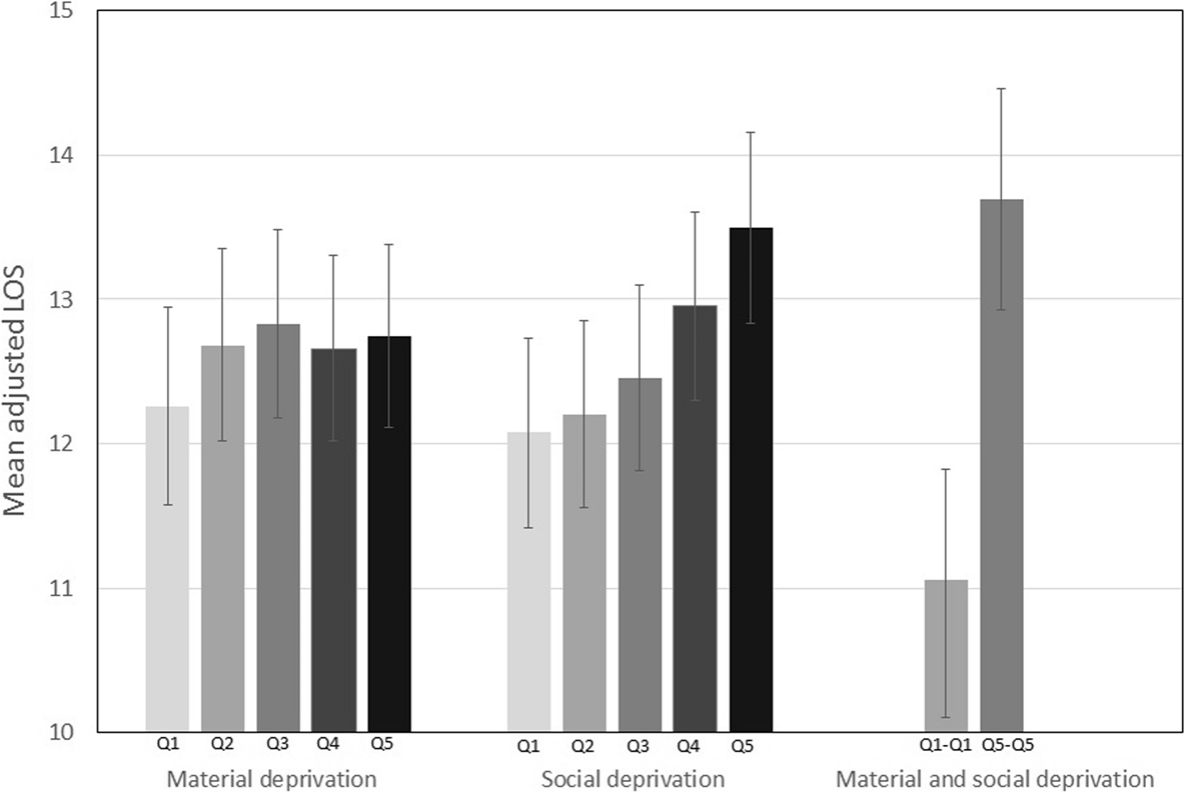
Adjusted mean acute care length of stay according to material and social deprivation

Mean LOS for the whole study population was 13.5 days. After risk adjustment, patients in the highest quintile of material deprivation had an LOS 0.5 days longer than those in the lowest quintile (Fig. 1, Table 2). However, LOS did not increase over the first four quintiles of material deprivation. Mean LOS increased with every quintile of social deprivation and was 1.4 days longer for patients in the highest quintile of social deprivation compared to those in the lowest quintile. The increase in LOS was even greater for patients in the highest quintiles of material and social deprivation who had a hospital stay on average 2.6 days longer than patients in the lowest quintiles. When analyses were stratified for age, increases in LOS associated with social deprivation and social-material deprivation were greater for elderly patients than their younger counterparts (Table 2). However, unlike patients < 65, elderly patients suffering from high material deprivation did not have a longer LOS.

**Table 2:** Adjusted mean differences and 95 % confidence intervals (CI) in hospital length of stay (LOS) according to socio-economic deprivation by age

Socio-economic status	N (%)	Adjusted^a^ difference in mean LOS (95 % CI)		
		All patients	<65 years	≥65 years
Material deprivation				
Q1	8639 (13.2)	1.00	1.00	1.00
Q2	11,229 (17.2)	0.43 (0.04-0.82)	0.36 (−0.05-0.77)	0.35 (−0.35-1.05)
Q3	13,439 (20.5)	0.57 (0.20-0.95)	0.57 (0.17-0.97)	0.33 (−0.35-1.02)
Q4	15,007 (22.9)	0.41 (0.03-0.78)	0.37 (−0.02-0.77)	0.23 (−0.45-0.90)
Q5	17,172 (26.2)	0.49 (0.12-0.87)	0.70 (0.32-1.09)	−0.01 (−0.71-0.69)
Trend p-value		0.048	0.006	0.7
Social deprivation				
Q1	11,551 (17.6)	1.00	1.00	1.00
Q2	13,108 (20.0)	0.13 (−0.22-0.47)	0.13 (−0.21-0.48)	0.15 (−0.52-0.83)
Q3	13,932 (21.3)	0.38 (0.03-0.72)	0.46 (0.12-0.81)	0.38 (−0.29-1.04)
Q4	13,648 (20.8)	0.87 (0.53-1.22)	0.69 (0.33-1.04)	1.08 (0.43-1.73)
Q5	13,247 (20.2)	1.42 (1.06-1.77)	1.22 (0.86-1.59)	1.70 (1.04-2.36)
Trend p-value		<0.0001	<0.0001	<0.0001
Material and social deprivation				
Q1-Q1	1379 (2.1)	1.00	1.00	1.00
Q5-Q5	3535 (5.4)	2.64 (1.77-3.50)	2.27 (1.41-3.13)	3.22 (1.54-4.90)
*P*-value		<0.0001	<0.0001	<0.0001

LOS length of stay, *Q* quintile

^a^Adjusted for age, gender, number of comorbidities, mechanism of injury, Maximum Abbreviated Injury Scale score, body region of the most severe injury, Glasgow Coma Scale score, systolic blood pressure, respiratory rate, and transfer status

### Sensitivity analyses

Modelling deaths (*n* = 3012, 4.6 %) by attributing an LOS of 120 days led to a slightly smaller difference in LOS for patients in the highest quintile of material, social and material-social deprivation (mean adjusted difference = 0.32, 1.21, and 2.38 days, respectively). Excluding observations with missing physiological data led to a slightly larger difference in LOS for patients in the highest quintile of material deprivation (mean adjusted increase = 0.64) but a slightly smaller difference for patients in the highest quintile of social and material-social deprivation (mean adjusted difference = 1.08 and 2.31 days, respectively). Overall, 6.7 % of patients had more than one consecutive hospital stay related to their injury. Mean total LOS was 1.2 days longer than mean index LOS (14.6 days). The SES-LOS association remained unchanged when total LOS was modelled over index LOS.

## Discussion

In this multicenter cohort study in an integrated and universal access trauma system, patients admitted for injury in the highest quintiles of social and material deprivation had an acute care stay on average 2.6 days or 24 % longer than patients in the lowest quintiles. The observed difference in LOS was more pronounced for social deprivation (1.4 days on average) than for material deprivation (0.5 days on average) and was even greater for patients suffering both material and social deprivation.

The association between SES and hospital LOS has been inconsistently documented in the literature. Increases in LOS for increasing material deprivation have been reported for general admissions in inclusive healthcare systems [7] but not in for-profit systems [38]. Increases have also been observed for US stroke admissions [8] and admissions for patients <65 years of age with a primary diagnosis of pneumonia in Canada [10] but not for patients admitted for traumatic brain injury in the US [39] or geriatric patients admitted for pneumonia [9]. However, all of these studies were based on only one dimension of material deprivation, median neighborhood income. Few studies have evaluated the association between social deprivation and LOS but one study in elderly pneumonia admissions observed a longer LOS for patients living alone [9]. We identified four studies that used multifactorial composite indices of social/material deprivation to evaluate the association between SES and hospital LOS. Longer LOS was observed for high deprivation categories in coronary artery bypass [40], chronic obstructive pulmonary disease [41] and psychiatric [11] admissions but not for stroke patients [42]. However, these studies did not report results separately for social and material deprivation and no stratification was conducted for age.

Further analyses are needed to evaluate whether prolonged hospital stays among patients suffering high material and in particular, social deprivation are inappropriate and to identify factors that may explain the observed difference. However, research suggests that up to 80 % of unnecessary hospital days are due to delays accessing government-funded long-term care facilities or community aid [10, 43, 44]. Increased LOS in patients with low SES would thus be consistent with lack of access to natural caregivers (social deprivation) [10, 45, 46] and lack of material resources to pay for private post-discharge care (material deprivation) [7]. The weaker association for material deprivation observed in this study may be due to the presence of a universal healthcare system and a strong social safety net in Canada. The lack of association between material deprivation and LOS in elderly patients has been observed elsewhere [9, 38] and may be due to the greater presence of community care and long-term care facilities for elderly patients than their younger counterparts.

### Strengths and limitations

This study population is representative of moderate to major trauma admissions in the province (population based) because it includes admissions to all trauma centers (level I to level IV) in a fully integrated system. Previous research has shown that over 90 % of patients hospitalized for major trauma in the province are treated within the trauma system [47]. In addition, the trauma registry used in this study is audited periodically to ensure data quality and audit results suggest high data accuracy (only 76 errors in 65 data fields × 80 patient files; data not published). Furthermore, our study used a comprehensive measure of SES based on six indicators of social and material deprivation.

However, this study does have limitations which should be considered in the interpretation of results. First, SES was defined using an ecological measure instead of an individual measure, which may have led to an underestimation of the SES-LOS association [31]. However, one advantage of ecological measures is that they take account of the living environment and neighborhood influences [48]. Second, we excluded 9 % of patients due to missing information on SES. Patients with missing SES had a longer mean LOS (14.8 days) than those included in the study (13.5 days). SES was mainly missing in low-density rural areas and areas where more than 15 % of the total population live in institutions or collective households [31]. Considering these patients are likely to have higher levels of material and social deprivation and worse outcomes than those included, SES-LOS associations may again have been underestimated. Third, approximately 10 % of major trauma cases are treated in non-designated centers in the province and so were not included in this study [47]. However, we have no reason to believe that the association between SES and LOS would not be observed in trauma patients treated outside the system. Fourth, previous research suggests that low SES is associated with an increased risk of unplanned readmission [12, 14, 49]. We anticipate that not accounting for these additional acute care days may have led to an underestimation of the SES-LOS association in our study. Fifth, poor data quality, a common problem in retrospectively-collected data, may have led to residual confounding due to unmeasured severity. However, misclassification of injury severity would only explain observed differences in LOS if injury severity is more frequently underestimated in patients with high social deprivation, which we consider unlikely. Finally, results may be subject to survival bias because deaths were excluded from analysis. Indeed, if low SES is associated with a higher probability of death following admission for injury, the exclusion of fatalities may have led us to underestimate the association between SES and LOS. However, an analysis with deaths included and attributed an arbitrarily long LOS led to similar results.

#### Potential policy implications

The results of this study may have important implications for improving resource use and outcomes for patients admitted following injury that suffer from material and social deprivation. Indeed, given the volume of injury hospitalizations in Canada (205,000 per year) [17] and an average cost per acute care bed of CAN$ 360.95 [6], reducing mean LOS to that observed in the lowest quintiles of material and social deprivation would lead to savings of approximately 513,282 (13 % of 185,000 bed days) or CAN$ 185 million per year in Canada. In addition, given the negative consequences of prolonged LOS in terms of adverse events including hospital-acquired infections and functional decline [3–5], interventions designed to reduce the influence of social disparities on LOS may have the potential to improve patient morbidity and mortality. These interventions could include comprehensive patient risk assessment, early discharge planning and patient education. These interventions, along with effective communication and cooperation between health and social workers as well as hospital and community care systems, have been shown to decrease unnecessary acute care days [6]. Results also suggest that SES should be taken into account in hospital resource allocations to avoid unfairly penalizing hospitals in areas of high socioeconomic deprivation [7].

## Conclusion

Patients admitted for injury suffering from material and particularly social deprivation may have longer mean LOS than their counterparts in a universal healthcare setting. Further research is needed to identify factors contributing to possibly unnecessary acute care days in these patients. Results suggest that consideration of SES in discharge planning and community care attribution may lead to reductions in LOS, which would in turn improve resource use and outcomes for injury admissions.

## Abbreviations

LOS: Length of stay
SES: Socio-economic status
MAIS: Maximum abbreviated injury scale score
GCS: Glasgow coma scale score
SBP: Systolic blood pressure
RR: Respiratory rate
